# Haemostatic Role of TachoSil Surgical Patch in Cardiac Surgery

**DOI:** 10.5681/jcvtr.2014.020

**Published:** 2014-06-30

**Authors:** Alireza Alizadeh Ghavidel, Yalda Mirmesdagh, Niloufar Samiei, Maziar Gholampour Dehaki

**Affiliations:** ^1^Heart Valve Disease Research Center, Rajaie Cardiovascular Medical & Research Center, Iran University of Medical Sciences, Tehran, Iran; ^2^Rajaie Cardiovascular Medical & Research Center, Cardiac Surgery Department, Iran University of Medical Sciences, Tehran, Iran

**Keywords:** Bleeding, Blood Transfusion, Cardiac Surgery, Hemostasis

## Abstract

***Introduction:*** Excessive bleeding presents a risk for the patient in cardiovascular surgery. Local haemostatic agents are of great value to reduce bleeding and related complications. TachoSil (Nycomed, Linz, Austria) is a sterile, haemostatic agent that consists of an equine collagen patchcoated with human fibrinogen and thrombin. This study evaluated the safety and efficacy of TachoSil compared to conventional technique.

***Methods:*** Forty-two patients scheduled for open heart surgeries, were entered to this study from August 2010 to May 2011. After primary haemostatic measures, patients divided in two groups based on surgeon’s judgment. Group A: 20 patients for whom TachoSil was applied and group B: 22 patients that conventional method using Surgicel (13 patients) or wait and see method (9 cases), were performed in order to control the bleeding. In group A, 10 patients were male with mean age of 56.95±15.67 years and in group B, 9 cases were male with mean age of 49.95±14.41 years. In case group 70% (14/20) of the surgeries were redo surgeries versus 100% (22/22) in control group.

***Results:*** Baseline characteristics were similar in both groups. In TachoSil group 75% of patients required transfusion versus 90.90% in group B (P=0.03).Most transfusions consisted of packed red blood cell; 2±1.13 units in group A versus 3.11±1.44 in group B (P=0.01), however there were no significant differences between two groups regarding the mean total volume of intra and post-operative bleeding. Re-exploration was required in 10% in group A versus 13.63% in group B (P=0.67).

***Conclusion:*** TachoSil may act as a superior alternative in different types of cardiac surgery in order to control the bleeding and therefore reducing transfusion requirement.

## Introduction

Excessive bleeding is a serious complication during cardiovascular surgery and is related to significant morbidity and mortality, increased duration and costs of surgery and prolonged postoperative hospital stay.^1^ Bleeding is mostly common and significant in arterial bleeding with difficult access as in case of a redo surgery, aortic root reconstruction and in old age group. In some of these patients the significant amount of bleeding cannot be stopped using haemostatic sutures and oozing from suture lines will continue despite the treatment for coagulation disorder. Therefore, there are various types of devices and drugs such as liquid fibrin sealants, oxidised cellulose fleece, cotton gauze and synthetic glues with different types of conventional surgical methods like compression, ligation, clipping and electrocautery, to promote homeostasis.

TachoSil (Nycomed, Linz, Austria) is a sterile, haemostatic agent that consists of an equine collagen patch coated with human fibrinogen and thrombin.^1^ TachoSil is a developed form of TachoComb that does not contain aprotinin or bovine thrombin .An increase in the number of reported cases of immunologic responses and renal failure, caused by the TachoComb^2^ has resulted into the new version of the product TachoSil. By the aid of moisture on application, this product is activated, providing adherence to the resection surface which results into homeostasis in 3 to 5 min of compression.^1^

Although several studies have been previously conducted in order to show the efficacy and safety of TachoSil in patients undergoing lung surgery^3-5^, liver resection^6^, splenectomy^7^, and different fields of surgery^8^, there are few articles which are related to the use of TachoSil in cardiovascular surgery. The aim of this study is to assess the safety and efficacy of this product during cardiac surgeries.

## Methods and materials

After approval of the study protocol was granted by the institutional review board and written informed consent was obtained from each patient, 42 patients scheduled for elective valve surgery (32), coronary artery bypass grafting (CABG) (5) or combined procedure Bentall and CABG (3), CABG and valve repair (1), pulmonary thromboendartrectomy (PTE) and CABG (1), were entered to this cohort study from August 2010 to May 2011. At the end of the surgeries and during homeostasis phase, all patients who had insidious bleeding from suture lines or oozing from surgical planes despite of no coagullopathy and meticulous suturing, were divided into two groups based on the surgeon’s judgment. As for group A; 20 patients, TachoSil patch (Nycomed, Austria, GmbH) was used and for group B; 22 patients, the conventional technique: using Surgicel (Ethicon, Johnson & Johnson, medical limited 2008, Switzerland) (13 patients) or wait and see method (9 cases), were performed. The mean age of patients in group A was 56.95±15.67 and for group B it was: 49.95±14.41. The demographic data and basic characteristics of the patients have been shown in Table 1. In group A patients 70% (14/20) of the surgeries were redo surgeries as for group B this was 100%( 22/22). All the patients were evaluated during the operation and hospitalization time for total blood loss volume, re-exploration requirement in order to fix the tamponade, blood and blood products’ transfusion requirement and post–op morbidities and mortalities.

### Surgical technique

For the patients in group A, after protamine administration based on activated clotting time (ACT) and correction of underlying coagullopathy using blood products when needed, the mediastinal irrigation by NS 0.9% was done, afterwards according to the bleeding site area, TachoSil patch was trimmed and applied on the site and with the help of gauze, gentle pressure was performed on the site for 3 minutes. The site of bleeding was checked by taking away the gauze. This technique was performed for the second time if the bleeding had become less but there was a leak from the site. The sternum was closed after homeostasis. In addition, for the patients who had a few amount of bleeding after applying the technique twice, the wait and see method was performed. None of the patients needed mediastinal packing due to persistent bleeding. As for group B patients, this technique was performed with the help of Surgicel. The hemoglobin (Hgb) level was maintained above 8 gm/dl for the patients who were not considered high risk with age of ≤60 years old, as for high risk patients [patients with left ventricular dysfunction and/or Chronic Obstructive Pulmonary Disease‏ (COPD)]the Hgb level was maintained above 10 gm/dl.

### Statistical analysis

Statistical analyses were performed with SPSS software version 15. Clinical data are expressed as mean±standard deviation. Differences were analyzed with paired and independent student’s T-test for the values of a scaling term and Pearson’s Chi-Square test for nominal values. Mann Whitney U test was used for comparing the values without normal distribution between two groups. A value of P<0.05 was considered statistically significant.

## Results

The baseline characteristics were similar between groups. Among the study groups there were only 3 patients who required TachoSil patch twice in order to control the bleeding. Sixty seven percent of the patients received pre-operative antifibrinolytic agents that included tranexamic acid or aminocaproic acid and the two groups of our study were similar in receiving the pre-op antifibrinolytic (P=0.12). The amount of need for transfusion in operation room, during the surgery and in ICU were significantly different between our study groups. This was 75% for group A versus 90.90% for group B (p=0.03). Also the mean unit of packed red blood cell (PC) that each group received was different statistically, group A received 2±1.13 units of PC as group B received 3.11±1.44 (P=0.01). However, the two study groups were similar in receiving fresh frozen plasma (FFP) and platelets (Plt). Table 2 shows the differences between two groups in receiving blood components and blood transfusion. There were no significant differences between two groups regarding intra operative, first day and total post operative mediastinal bleeding. The mean volume of intra operative blood loss before closing the sternum was 264.29±439.15 cc in group A versus 450.00±187.08 cc in group B (P= 0.15). The mean volume of bleeding in 24 hours after the surgery was 341.82±337.42 cc in group A as this was 537.37±391.68 cc in group B (P=0.09). Also the mean total volume of blood loss after the operation and before extracting the chest drainage in group A was 656.67±434.03 cc versus 1136.67±1300.81 cc in group B (P=0.29). The Intra-operative characteristics have been shown in Table 2. A total of 21 events were reported, 10 events in 20 (50%) TachoSil-treated patients and 11 events in 22 (50%) standard-treated patients with no statistical difference. The most reported events were non-cardiac problems; TachoSil, n=9; standard treatment, n=10 and the most cardiac events were supraventricular arrhythmias (including atrial fibrillation; 1 case in each group). All of which are known complications of the surgical procedure and underlying disease. Hypersensitivity reactions or TachoSil related infectious complications were not reported. There was no significant difference regarding the incidence of mortality between groups. Five death occurred, three as a result of sepsis with multi-organ failure (one TachoSil patient and two in group B patients) and two as a result of myocardial infarction (group A) and respiratory failure (group B). Re-operation was required in 1 patient (5%) in group A and 2 (9.09%) in group B (P=0.06). The need for re-exploration was not related to the study treatment or target area in any patient.

**Table 1:** Baseline characteristics of the patients

	**Group A** **(n=20)**	**Group B** **(n=22)**	**P value**
Age ( Years, Mean± SD)	56.95±15.67	49.95±14.41	0.14
Male/Female (n)	10/10	9/13	0.07
BW ( Kg, Mean±SD)	71.80±13.51	68.67±21.04	0.57
BSA (Mean±SD)	1.72±0.17	1.59±0.17	0.02
**Past medical history**			
Diabetes mellitus (%)	20	13.63	0.58
Hypertension (%)	30	18.18	0.36
Coagulopathy (%)	5	No	0.28
Renal Failure (%)	NO	No	−
Liver Failure (%)	NO	No	−
COPD (%)	5	No	0.28
**Drug history**			
Plavix (last 24 h %)	NO	4.5	0.33
A.S.A (last 72 h % )	55	31.81	0.12
Pre-op LVEF (Mean ±SD)	44.55±6.50	48.26±5.28	0.11
Pre-op Hct ( Mean ±SD)	37.88±4.48	36.59±6.86	0.49
Pre-op Plt ( Mean ±SD)	205444.44±88593.73	296190.48±44782.56	0.37
Pre-op PT (Mean ±SD)	15.53±3.44	16.42±4.08	0.39
Pre-op PTT ( Mean ±SD)	41.71±16.12	36.06±12.84	0.18
Pre-op INR (Mean ±SD)	1.41±0.49	1.53±0.74	0.74
**History of operation**			0.15
No	2 (10)	No	
OMVC	2 (10)	3 (13.63)	
MVR	4 (20)	7 (31.81)		
CABG	2 (10)	2 (9.09)	
AVR+MVR	1 (5)	3 (13.63)	
MVR+TVR	1 (5)	No	
MVR+TVR+AVR	1 (5)	No	
Bentall	2 (10)	No	
TFTC+PVR	1 (5)	1 (4.54)	
AVR+VSD repair	1 (5)	1 (4.54)	
AVR+CABG	1 (5)	1 (4.54)	
PVR+AVR+TFTC+ASD repair	No	1 (4.54)	
OMVC+MVR	1 (5)	2 (9.09)	
OMVC+AVR	1 (5)	1 (4.54)	
**Operated Procedures (%)**		0.21
CABG	15		9.09
Valve surgery	55		81.81
CABG+Valve surgery	10		9.09
Aorta surgery	15		No
CABG+ PTE	5		No

BW: Body Weight, BSA: Body Surface Area, COPD: Chronic Obstructive Pulmonary Disease, LVEF: Left Ventricular Ejection Fraction, Hct: Hematocrite, Plt: Platelet, PT: Prothombin Time, PTT: Partial Thrombin Time, INR: International normalization ratio, OMVC: Open Mitral Valve Commissurotomy, MVR: Mitral valve replacement, CABG: Coronary artery bypass grafting, AVR: Aortic valve replacement, TVR: Tricuspid valve replacement, TFTC: Tetralogoy of Fallot Total Correction, PVR: Pulmonary valve replacement, ASD: Atrial septum defect, VSD: Ventricular septum defect, PTE: Pulmonary thromboendartectomy

**Table 2:** Intra-operative Characteristics

	**Intra Operation**	**Group A (n=20)**	**Group B (n=22)**	P value
Transfusion Need (%)	75	90.90	0.03
No. PC (Mean±SD)	2±1.13	3.11±1.44	0.01
No. FFP (Mean ±SD)	2.78±1.98	1.70±2.02	0.09
No. PLT (Mean±SD)	1.50±2.09	2.26±2.05	0.20
Operation Time (Mean±SD)	217.40±70.33	213.33±88.36	0.88
CPB Time (Mean±SD)	105.12±54.18	114.90±57.05	0.59
AOX Time (Mean±SD)	54.88±25.04	62.16±31.76	0.45

PC: Packed Cell, FFP: Fresh Frozen Plasma, PLT: Platelet, CPB: Cardio pulmonary bypass, AOX: Aortic cross clamping

## Discussion

Excessive bleeding is a serious complication of cardiovascular surgery and is associated with significant morbidity and mortality.^1^ Bleeding is mostly common in arterial bleeding with difficult access in case of redo surgeries, aortic root reconstructions and in old age group. In some of these patients the significant amount of bleeding cannot be stopped using haemostatic sutures. Therefore there are different types of devices to control the bleeding. One of them is TachoSil (Nycomed, Linz, Austria) which is a sterile, ready to use haemostatic agent. Although several studies have been previously done to show the efficacy and safety of TachoSil in patients with lung problems^3-5^, liver resection^6^ and splenectomy^7^ there are a few articles related to the use of TachoSil in cardiovascular surgery.^1^ In this study we evaluated different factors such as the amount of need for transfusion, PC, FFP, Plt and drainage. Our study revealed that the amount of need for FFP and Plt were lower in group A: the patients with TachoSil than group B: the patients with no TachoSil but these differences were statistically insignificant. Also the amount of need for transfusion and P.C were lower in group A than group B, with significant differences (P=0.03, P=0.01, respectively). Although the drainage volume was lower in group A than group B but this difference was not significant. Another result of our study was that there were no significant differences regarding operation time, CPB time and AOX time between our 2 groups of study which they all can lead to more platelets’ disruption and post–op bleeding yet the amount of need for transfusion was significantly lower in group A. The similar result in the study of Hass^9^ is seen in which they claimed that the use of the topical hemostant, TachoSil may have led to less need for blood component therapy. In our study only three patients in the group A needed applying TachoSil twice in order to achieve haemostasis but none of them needed re-exploration, as a result the success rate of TachoSil was high in order to control the bleeding. Also in our study no allergic reaction in surgical field or systemic reaction which were related to TachoSil were reported and also mediatinitis did not occur in neither of groups. Maisano et al.^1^ concluded in their study that TachoSil was significantly superior to standard haemostatic fleece material in order to obtain better and fast intra-operative haemostasis in cardiovascular surgery, TachoSil was safe and well tolerated. Although in this study the patients were not similar regarding the types and times of their procedures, it seems that TachoSil may act as a good alternative in order to control the non-surgical bleeding without inducing any post–op infectious or allergic complications in following cases:

1- Aortic surgeries:

- In aortic root surgeries with no specific source but continuous oozing from suture lines
- In patients with aortic dissection to support suture lines
- In elderly patients to support the suture lines

2- CABG cases:

- In patients suffering from minor bleeding from distal graft anastomosis especially in cases with deep coronary arteries who need to get their fat tissue or myocard dissected in order to gain access to coronary arteries
- In cases with extensive coronary enderctomy with long segment anastomosis with oozing from suture lines which in these cases an extra suture is more harmful than useful in order to control the bleeding due to tissue inconsistency
- In some coronary patients with small or poor run-off coronary arteries especially in diabetic patients with minor bleeding from the head and/or the heal of anastomosis which additional sutures can lead to narrowed anastomosis

3-Valve surgeries:

- In patients with severe Tricuspid regurgitation (TR) and enlarged right atrium with thin wall especially in redo surgeries in which control of bleeding from suture lines with the aid of extra sutures is hardly possible
- In cases suffering from Tricuspid valve disease and concomitant liver dysfunction with bleeding from suture lines in spite of managing the coagulative disorders

4-Others:

- In patients undergoing redo cardiac surgeries with de-epithialized cardiac surface because of the decortication and punctuated bleeding through the surface of the heart
- In cases with constrictive pericarditis who are undergoing pericardectomy and experiencing punctuated bleeding due to adhesion and inflammation

Therefore, As it mentioned before, TachoSil may act as an proper alternative when the surgeons are not able to control the oozing or punctuated bleeding in spite of performing the techniques such as conducting perfect sutures or fixing the coagulative disorders and for the time when surgeons have concerns about post-op mediastinal bleeding in ICU that leads to blood and blood component transfusion or temponade and as a result re-exploration.

## Limitations

The number of studied cases in this study is limited and they were divided in 2 groups based on the surgeon’s judgment, therefore selection bias cannot be ruled out.

## Acknowledgements

We thank Dr. Hooman Bakhshandeh for statistical analyses and Mrs. Fatemeh Ebrahimpour for collecting the required data during this study at Rajaie Cardiovascular Medical and Research Center.

## Ethical issues

This study was approved by our local Ethics Committee.

## Competing interests

Authors declare no conflict of interest in this study.
